# Differences in lung cancer screening outcomes and follow-up by patient, provider and place-based characteristics in Missouri and Illinois: A cross-sectional study

**DOI:** 10.21203/rs.3.rs-7745656/v1

**Published:** 2025-10-03

**Authors:** Akila Anandarajah, Vaishnavi Mamillapalle, Benjamin Bowe, Isaac Che Ngang, Alex Ramsey, Beryne Odeny

**Affiliations:** WashU Medicine; WashU Medicine; WashU Medicine; Missouri Baptist Medical Center, Barnes-Jewish Healthcare; WashU Medicine; WashU Medicine

## Abstract

**Background:**

Lung cancer remains the leading cause of cancer-related mortality in the United States, yet disparities in lung cancer screening (LCS) outcomes exist and remain understudied, particularly in the Midwestern region. Our objective was to investigate disparities in lung radiology outcomes and follow-up care based on patient, provider, and place-based characteristics (i.e., area deprivation index; ADI).

**Methods:**

This cross-sectional study used data from the LCS program at Siteman Cancer Center (SCC) in St. Louis, Missouri, from January to December 2023. SCC’s catchment area includes 82 counties in Missouri and Illinois; approximately 15% of the population reside in a rural zip code, and 29% reside in medically underserved areas 80% are White. The study included 1,946 individuals aged 50–80, meeting LCS eligibility criteria based on smoking history and age. Lung radiology findings were assessed as primary outcomes, and timely follow-up adherence (i.e., return for follow-up visit) was analyzed among patients with high-risk findings (Lung-RADS 3 [”Probably Benign”] and 4 [”Suspicious”]) from January to June 2023, requiring follow-up by December 2023. Multivariable logistic regression was conducted, adjusting for patient and provider characteristics and ADI.

**Results:**

Of the 1,946 individuals who accessed LCS, 57% were Black, 41% White, 1% Asian, and 1% of another race; 54% were male and 46% female. Lung findings were classified as “probably benign/suspicious” (high risk) for 14%. Annual visits were associated with higher likelihood of high-risk scores compared to baseline visits (AOR = 2.10 (1.40–3.15); p < 0.001). Racial differences were noted in the association between provider type and lung outcomes. Among White individuals only, specialist compared to primary care provider referral was associated with increased odds of being high risk (AOR = 1.58 (1.00–2.50); p = 0.048). Sex, insurance, smoking status, park-years and ADI were not associated with lung radiology outcomes. Timely adherence to return follow-up visit among high-risk patients was suboptimal, with only 20.0% returning within 3–6 months for their repeat LCS. Individuals residing in moderate ADI (distress) areas were less likely to have timely follow-up (for high-risk findings) compared to those in high distress (AOR = 0.393, 95% CI = 0.155–1.001, p = 0.050). There were no differences by sex, insurance, smoking status, and pack-years for being classified as high risk.

**Conclusions:**

SCC’s LCS program successfully captured Black populations and individuals from highly distressed areas, in a predominantly White catchment area. There were no observable race disparities in timely follow-up of high-risk findings, reflecting progress toward equity in access and outcomes. Place-based disparities in follow-up were observed that warrant further characterization and risk assessment to improve follow-up of patients undergoing LCS.

## Introduction

Lung cancer is the leading cause of cancer-related death and second most common cancer in the United States.^1^ Lung cancer screening (LCS) enables earlier detection of lung cancer, when the disease is more responsive to treatment and associated with better clinical outcomes.^2,3^ In clinical trials, low-dose computed tomography (LDCT) scans significantly reduced lung cancer mortality by 20%.^4–6^ Additionally, prior research found that more than half of lung cancers were detected at repeat annual screenings; therefore, adherence to ongoing follow-up screenings is critical for secondary cancer prevention.^7^

Based on this evidence, the US Preventive Services Task Force (USPSTF) recommends annual low-dose CT scans for high-risk individuals, identified as adults aged 50–80 with a history of smoking at least 20 pack-years, who either currently smoke or quit within the last 15 years.^8^

Despite evidence-based guidelines and implementation efforts, LCS usage remains low. Of the 8.5 million current and former smokers eligible for LCS, only 18% have received screening,^9^ and adherence to annual re-screening is also low (37–51%).^10^ Further, prior research found that only 12% of primary care physicians ordered LCS for any eligible patients in the past year. ^11^ This reflects multilevel cancer care gaps at the physician and patient levels. Physician ordering of LCS is insufficient, likely due in part to lack of patient-specific data to properly identify and indicate the urgency of LCS, and lack of knowledge about LCS effectiveness or how to discuss benefits and risks with patients.^12,13^ Additionally, accessing and using guideline-based care is challenging among patients with lower socioeconomic status, less insurance coverage and lower levels of education.^12,13^

In addition, women and Black men are more likely to be ineligible to meet the LCS criteria, being diagnosed at earlier age.^14,15^ Additionally, people of low socioeconomic status may be less likely to be eligible for LCS.^16^ The USPSTF explicitly identified a need for future research to determine whether LCS benefits differs when expanded to more diverse community settings of 1) racial and ethnic minorities, 2) socially and economically disadvantaged groups with higher smoking rates and lung cancer incidence, and 3) more women being screened.^5,8^ LCS radiology findings and follow-up by patient demographics in a clinical setting are under-explored, especially in the Midwestern United States.^5^

**Figure 1:**
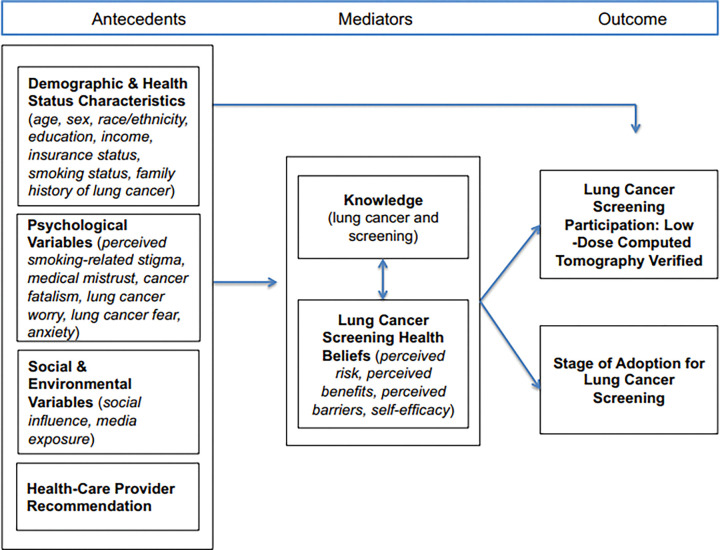
Conceptual Model of Lung Cancer Screening Participation ^17^

This paper will examine disparities associated with LCS findings and follow-up care by patient (e.g., race, sex), referring provider (e.g., specialty), and place-based (e.g., area deprivation index, rurality) factors in Missouri and Illinois. We leverage the *Conceptual Model for Lung Cancer Screening Participation* (Fig. 1) which illustrates the multilevel factors at patient, provider, and socio-environmental levels that are associated with participation in lung cancer screening uptake and adherence.^17^ To our knowledge, few studies have investigated LCS radiology findings—captured as the Lung CT Screening Reporting and Data System (Lung-RADS) scores—by these patient, provider, and place-based factors to expose and further characterize established inequities in access and outcomes of LCS. We aim to evaluate differences and disparities in lung radiology findings and follow-up care by patient, provider, and place-based factors in Missouri and Illinois.

## Methods

### Study Design, Source, and Preparation

We conducted a cross-sectional analysis using electronic health record data collected from January to December 2023 for the Siteman Cancer Center’s (SCC) LCS program at Barnes-Jewish Hospital in St. Louis, Missouri. The study involved 1,946 adults aged 50–80 who met LCS eligibility criteria according to USPSTF guidelines, which included a minimum of 20 pack-year smoking history and either being a current smoker or having quit within the past 15 years. SCC is a National Cancer Institute-designated Comprehensive Cancer Center that provides advanced cancer prevention, screening, diagnosis, and treatment services. It serves a diverse population from the St. Louis metropolitan region, extending its reach to urban and rural communities across eastern Missouri and southern Illinois. The racial distribution of the hospital’s catchment is 79.5% non-Hispanic White, 13.7% Black, 2% Asian, and 4.8% other groups. Approximately 3% of the population identifies as Hispanic/Latino ethnicity. SCC also plays a significant role in addressing healthcare needs across a wide socioeconomic spectrum, including medically underserved (29% of the population) and high-risk populations, and 15% reside in a rural zip code.

### Data Elements

Our dataset encompasses patient demographics, smoking history, Lung-RADS scores, insurance provider type, physician type, and indications and frequency of LCS screening. Missing pack-years data were imputed using the mean value of documented smoking history (pack-years) among all eligible individuals in the cohort, preserving the overall distribution, classification of Lung-RADS categories, standardization of smoking status, and categorization of insurance types as Medicare, Medicaid, private, or unknown.

In addition to patient-level clinical data collected from January to December 2023, we incorporated place-based variables sourced from external datasets. Specifically, Area Deprivation Index (ADI) scores—used to characterize socioeconomic disadvantage—were obtained from data last updated in 2020 by the Center for Health Disparities Research.^18,19^ ADI is a validated metric originally developed by the Health Resources & Services Administration (HRSA) and ranks neighborhoods at the Census block group level based on income, education, employment, and housing conditions. We calculated mean ADI ranks at the ZIP code level and categorized socioeconomic deprivation as high deprivation (above 70), moderate deprivation (40–69) and low deprivation (1–39).

Data on place-based characteristics (i.e., urban, rural), categorized by zip codes, were collected from the United States Department of Agriculture (USDA) database—most recently updated on August 17, 2020.^20^ ZIP codes were categorized as urban (secondary RUCA [Rural-Urban Commuting Area] scores 1–3) or rural (scores 4–9). For ZIP codes with a secondary RUCA value of 99, we conducted a manual search to determine whether they were urban; otherwise, we labeled them as “Unidentified.”

Provider specialization was determined by manually assigning a clinical specialty to each provider based on their name. Providers with backgrounds in family medicine, internal medicine, physician assistant, and nurse practitioner were grouped under the category of primary care providers (PCPs). All others, including those not explicitly categorized, were grouped under specialists. This classification allowed analysis comparing outcomes between patients seen by PCPs versus specialists.

### Outcomes

The Lung-RADS is used as a standardized system to report LCS results. Lung-RADS are graded in four categories ranging from negative (category 1) to suspicious (category 4). Higher scores indicate that nodules were found in the lungs that have a higher probability of being lung cancer.^21^ Lung-RADS scores that are probably benign (3) or suspicious (4) (high-risk) necessitate returning for sooner follow-up LCS than the standard one-year timeframe.

Our outcomes of interest were the Lung-RADS score; for those with high-risk Lung-RADS findings, we were interested in whether they had one or multiple visits (i.e., follow-up) within 2023. We stratified results by race, sex and ADI. Individuals with Probably Benign Lung-RADS findings should be scheduled to undergo repeat LCS in 6 months, and those with Suspicious Lung-RADS findings should return at least within 3 months, while those with negative or benign findings can wait until a year for their next screen (14).

We reviewed patient appointment records to identify individuals who required follow-up LCS within 3–6 months based on their Lung-RADS scores, the primary outcome measure for LCS. For the purposes of this study, we defined high-risk patients as those with a Lung-RADS score of 3 or 4 (i.e., probably benign or suspicious findings requiring closer surveillance), and low-risk patients as those with a Lung-RADS score of 1 or 2 (i.e., negative or benign findings). We identified the earliest recorded LCS appointment between January 1 and June 30, 2023, and classified patients with high-risk scores as requiring follow-up within 3–6 months. Focusing on patients with such scores in the first half of 2023 ensured a sufficient 6-month window within our dataset to observe their return for follow-up imaging. We examined records to determine whether the same patients had a subsequent, follow-up LCS visit in 2023. We categorized high-risk patients who completed more than one visit in 2023 as “Multiple Visit” patients, and high-risk patients who had only one visit in 2023, without any follow-up visit, as “Single Visit” patients.

### Statistical Analysis

We conducted descriptive analyses to summarize patient, provider, and place-based characteristics. We calculated frequencies and percentages for categorical variables and mean and standard deviation for pack-years. Age was dichotomized as below and above 65 years, reflecting the Medicare eligibility threshold, which may influence insurance coverage and access to LCS. Multivariable logistic regression was conducted to identify predictors of Lung-RADS outcomes, with the analysis stratified by race and ADI. Statistical significance was assessed at a two-sided alpha level of 0.05. We adjusted for age, smoking status (current, former) and pack-years, insurance type (Medicaid, Medicare, private, unknown), and provider type (e.g., PCP, specialist).

Under race, we removed Asian and “Other race” categories from inferential analyses due to very low numbers.

### Handling Missing Data

To ensure the integrity and completeness of the analytical dataset, we addressed missing data systematically. First, 20 records lacking ZIP code information or with ZIP codes outside Illinois (IL) and Missouri (MO) were excluded across the combined patient-level clinical and place-based datasets (i.e., SCC database, Area Deprivation Index, and USDA rurality data) to avoid inconsistencies in location-based analyses.

For continuous variables such as smoking history (pack-years), missing values were imputed using the mean value calculated from documented cases within the eligible cohort. This approach preserved the distributional properties of the variable while minimizing bias. Categorical variables were handled through logical imputation or default classification to maintain consistency. For example, missing values in the socioeconomic distress index (ADI) were assigned to the “Low Distress” category to reduce misclassification.

All analyses were conducted using SAS Version 9.4 (SAS Institute Inc., Cary, NC).

## Results

### Population Characteristics

Among the 1,946 individuals (Table 1), 1880 individuals with data, were included in inferential analysis, 53.9% were under 65 years old, and 46.1% were 65 or older. The cohort consisted of 53.6% males and 46.4% females. Over half (56.7%) of the participants identified as Black, 40.9% as White, 1.2% as Asian, and 1.2% as other races.

Most patients had Benign (59.1%) and Negative (29.2%) LungRADS category, i.e., 88.3% had Low-risk scores. Patients in the probably benign and suspicious LungRADS category, were 6.1% and 5.5%, respectively, for a total of 11.7% with High-risk scores.

Most individuals were current smokers (71.1%), and 28.9% were former smokers. The average number of pack-years smoked was 36.51 (±10.95).The average number of pack-years smoked was 36.51 (±10.95). 52.1% underwent their first ever baseline LCS, while 47.9% attended a repeat annual LCS. A majority (98.3%) resided in urban areas, with only 1.7% from rural regions. Nearly half of the patients (48.8%) lived in areas with high distress, 25.0% in moderate distress, and 26.3% in low distress. In terms of insurance status, 53.1% were covered by Medicare, followed by nearly equal proportions with private insurance (23.0%) and Medicaid (22.9%), while 1.0% had unknown insurance. Most were seen by a PCP (85.0%), while 15.0% were seen by specialists.

### Unadjusted LCS Outcomes

We examined the unadjusted distribution of low-risk (Benign or Negative) and high-risk (Probably Benign or Suspicious) across characteristics using cross-tabulations and chi-square tests (Table 3). Overall, 88.3% of participants had low-risk findings, and 11.7% were categorized as high-risk.

#### Age group:

There was a significant association between age group and RADS category (OR = 1.34; 95% CI: 1.00–1.80; p=0.05). Participants aged ≥65 years were more likely to have high risk scores compared to those <65 years.

#### Sex:

There was no association between sex and RADS category (p=0.117)

#### Race:

There was no association by race (p=0.157), although White patients had a higher proportion of high-risk findings (12.9%) compared to Black patients (10.8%).

#### Insurance:

There was no association between insurance and RADS category approached ( private vs Medicaid, p=0.766; and private vs Medicare, p=0.124 ).

#### Screening indication (baseline vs annual):

There was a strong and significant association between screening indication and RADS category. Patients undergoing annual screening had a higher odds of high-risk scores compared to those getting their first, baseline screening (OR = 1.80; 95% CI: 1.33–2.42; p < 0.001).

#### Provider type:

Provider type was significantly associated with RADS category. Patients referred by specialists were more likely to have high-risk findings compared to those seen by PCPs (OR=1.33; 95% CI:1.00–2.07; p=0.047).

#### Smoking status:

There was no association between being a current or former smoker and RADS category (p= 0.85).

#### ADI:

There was no association between RADS category and different levels of area level deprivation. Compared to patients in low ADI areas, those in moderate ADI (p=0.278) and high ADI areas (p=0.817) did not differ significantly in their likelihood of being classified as high risk.

### Adjusted associations for LCS Outcomes

Among Black individuals, undergoing an annual (repeat) screen was significantly associated with high-risk scores (AOR = 1.85; 95% CI: 1.32–2.58; p < 0.001). Other variables—including age, sex, provider type, smoking status, insurance, ADI and pack-years—were not significantly associated with high-risk findings in Black individuals. Effect modification for race was significant (p=0.037).

Among White individuals, undergoing an annual (repeat) screen was significantly associated with high-risk lung-RADS scores (AOR = 2.10; 95% CI: 1.40–3.15; p < 0.001). Additionally, being seen by a specialist compared to a primary care provider was associated with increased odds of high-risk findings (AOR = 1.58; 95% CI: 1.00–2.50; p = 0.048). Other factors—including age, sex, smoking status, insurance, ADI and pack-years—were not significantly associated with high-risk findings in White individuals. Effect modification for race was borderline significant (p=0.058)

### Adherence to follow-up for high-risk findings

In Table 4, among the 220 patients identified as high-risk based on screening criteria, the majority (80.0%) completed only a single lung cancer screening (LCS) visit, while 20.0% had a timely return for the initial follow-up visit, i.e., within the recommended 3- to 6-month window.

The only variable approaching statistical significance was ADI. Individuals residing in moderately distressed areas had lower odds of timely adherence to initial follow-up visit compared to those in highly distressed areas (AOR=0.393; 95% CI: 0.155–1.001, p = 0.050). There was no difference in adherence to multiple visits comparing individuals residing in low vs moderate distress, or those in low vs high distress. Age, sex, insurance, race, smoking, screening indication, provider type and park- years.

## Discussion

This study evaluates Lung-RADS outcomes by patient, provider, and place-based characteristics from a LCS program based in MO and IL. Our analysis revealed statistically significant place-based and provider-specific (among White individuals) differences in LCS radiology findings. We observed that more than half of the patients were Black in this predominantly White catchment area, reflecting progress toward equitable access to the SCC program. Concerningly, we found that 70% of patients were current smokers, emphasizing a greater need for integration of tobacco cessation strategies into LCS programs, and potentially leveraging Lung-RADS findings as an opportunity to educate patients on smoking cessation. The emergence of point-of-care tobacco use treatment facilitated by electronic health records and embedded in routine oncology care visits may offer a targeted strategy to address this gap in care.^22^

Among White individuals only, those referred by specialists were more likely to have high-risk findings compared to those referred by PCPs. As this was not the case for Black individuals, it may signal differences in provider referrals by patient characteristics like race. We also found that high area distress was not associated poorer access to follow up care for high-risk findings. We found no significant variations in lung radiology outcomes or follow-up by sex, age, insurance, smoking status, and pack-years.

These findings suggest that concerted efforts—potentially including updated USPSTF 2021 guidelines and local cancer center strategies—to enhance access and care for marginalized racial groups may be yielding more equitable LCS outcomes. While there were no direct racial differences in lung-RADS, this does not alleviate concerns around the applicability of Lung-RADS classification to Black populations.^23^ A recent meta-analysis showed that Lung-RADS demonstrated significant heterogeneity between study populations in sensitivity and specificity,^24^ underscoring the need for more research on Lung-RADS performance in multiethnic populations. Given that Black individuals have higher lung cancer incidence than White individuals at a younger age, our findings of no association between race and likelihood of high-risk findings is interesting and may point to concerns raised about reliability of Lung-RADS in Black and other populations. This may also be explained by our high number of Black individuals attending LCS, as they may have been attending LCS more often. SCC serves a 20.5% minority population, yet 56.7% of participants screened were Black, highlighting the success of SCC’s community-oriented efforts to expand LCS access among the underserved.

With regard to provider referrals, when PCPs refer patients rather than specialists, this may encourage earlier LCS with less suspicious results. PCPs can play an important role in encouraging LCS,^25,26^ as individuals are much more likely to see a PCP rather than a specialist. Reserving time during a PCP appointment for shared decision making to discuss the risks and benefits of LCS could promote earlier uptake and more consistent adherence to detect lung cancers at more treatable stages.^27^ Training and supporting PCPs to refer their patients for LCS could lead to better patient outcomes.^28^ However, specialists may be more likely to see sick patients with high-risk scores.

We found no association between age and the outcomes. A prior study showed that older patients were more likely to be assigned high-risk results and found no associations with race or sex.^29^ The differences in findings may be attributed to the differing patient populations, as the prior study used a nationwide cohort from five different institutions located across the country.

We found 14.3% of patients had high-risk scores, which is similar to estimates provided by most other academic and community institutions throughout the United States, ranging from 11.4% in academic centers in North Carolina to 24.6% in an inner-city cohort including federally qualified health centers in Chicago.^30–33^ Additionally, when the Lung-RADS classification was retrospectively applied to the National Lung Screening Trial, they had a similar positivity rate of 13.6%.^34^ Two of these studies had a majority Black population, similar to the current study.^31,33^ This validates our findings and demonstrates that our over- and under-diagnosis rates are similar to other institutions.

Our finding that a higher incidence of suspicious findings among individuals returning for annual LCS underscores the importance of encouraging patients to receive regular LCS and establishing potential measures to track patients and incentivize retention in the LCS pathway. This facilitates earlier detection, enabling more treatment options and superior patient outcomes.^5^ LCS programs must monitor participants over time and minimize loss of follow-up to be most effective. Longitudinal tracking of multiple lung CT scans may also hold more data that could potentially be used to diagnose lung cancer earlier on.^35^

In our LCS program, most patients were Black in a predominantly White catchment area. This is noteworthy, as other programs have struggled with reaching Black patients and those of low socioeconomic status.^36^ People with low socioeconomic status are more likely to display fatalistic attitudes, seeing cancer as unpreventable, and are less likely to understand their cancer risk.^6,37^ To successfully serve these populations, targeted interventions should be developed to specifically address societal drivers of these disparities. This could be done by directly engaging patients to co-develop culturally competent interventions.^38^ SCC patient navigators also guide people through the LCS process. Navigators play an important role in educating patients about LCS, addressing patient concerns, reducing physician burden, and ensuring patients are not lost to follow-up.

Implementing navigators has been recommended, especially for vulnerable populations,^39^ and has been shown to increase LCS uptake in a randomized controlled trial.^40^ Sending targeted invitations to eligible participants in areas of high deprivation may also increase LCS uptake among these populations.^41,42^ We found individuals from high distress areas were more likely to return for the recommended follow-up for high-risk findings, possibly due to the influence of a SCC’s outreach strategies and robust patient navigation systems.

The strengths of this study include our robust sample size, diverse patient population, and range of multilevel factors (e.g., patient, provider, area-levels) evaluated. Being conducted in an urban academic center in the Midwest, findings may be generalizable to other similar settings with similar population characteristics. However, this study has several limitations. Our cohort included a low number of individuals not identifying as White or Black, including those who identified as Asian or of another race. It is possible that patients with high-risk findings who did not have timely follow-up visits moved to other locations or passed away. Although we adjusted for various factors the possibility of residual confounding remains due to the nature of cross-sectional study designs. Given the relatively small sample size of high-risk patients with category 3 or 4 findings (high-risk), we may have been underpowered to detect significant differences and associations. Another limitation is that follow-up adherence was only analyzed among patients with high-risk findings. Evaluation of follow-up adherence in low-risk( Lung-RADS 1 and 2) patients may have yielded different patterns and associated factors.

## Conclusions

### Results

#### Sex

There was no association between sex and RADS category (p = 0.117)

#### Race

There was no association by race (p = 0.157), although White patients had a higher proportion of high-risk findings (12.9%) compared to Black patients (10.8%).

#### Insurance

There was no association between insurance and RADS category approached ( private vs Medicaid, p = 0.766; and private vs Medicare, p = 0.124).

### Smoking status

There was no association between being a current or former smoker and RADS category (p = 0.85).

#### ADI

There was no association between RADS category and different levels of area level deprivation. Compared to patients in low ADI areas, those in moderate ADI (p = 0.278) and high ADI areas (p = 0.817) did not differ significantly in their likelihood of being classified as high risk.

## Conclusions

Our findings identify patient, provider, and place-based factors that may be associated with LCS outcomes and timely adherence to follow-up care. Further research is needed to understand the contribution and impact of these factors on Lung-RADS scores in risk assessment. SCC’s LCS program successfully captured individuals from highly distressed areas and Black populations, in a predominantly White catchment area. There were no observable race disparities in timely follow-up of high-risk findings, reflecting progress toward equity in access and outcomes. Place-based disparities in follow-up were observed that warrant further characterization and risk assessment to improve follow-up of patients undergoing LCS. Tailoring LCS criteria and risk assessment based on these insights can help clinicians improve lung cancer detection and follow-up efforts for high-risk patients.

### Ethical approval:

We obtained ethical approval from the Washington University Institutional Review Board and Siteman Cancer Center’s Protocol Review and Monitoring Committee. We received a waiver of consent for electronic record data extraction and were have been approved to obtain verbal consent from health providers.

## Figures and Tables

**Table 1:** Descriptive Characteristics

Variable	Category	N = 1946 (%)
Age	Above 65	897 (46.1)
	Below 65	1049 (53.9)
Sex	Female	903 (46.4)
	Male	1043 (53.6)
Insurance	Medicaid	445 (22.9)
	Medicare	1034 (53.1)
	Private	447 (23.0)
	Unknown	20 (1.0)
Race	Black	1104 (56.7)
	White	795 (40.9)
	Asian	24 (1.2)
	Other	23 (1.2)
Smoking Status	Current	1384 (71.1)
	Former	562 (28.9)
Indication for LCS evaluation	Annual LCS	933 (47.9)
	Baseline LCS	1013 (52.1)
Rural/Urban	Rural	34 (1.7)
	Urban	1912 (98.3)
Provider Type	PCP	1654 (85.0)
	Specialist	292 (15.0)
Lung-RADS Category	1 = Negative	568 (29.2)
	2 = Benign	1151 (59.1)
	3 = Probably Benign	119.0 (6.1)
	4 = Suspicious	108.0 (5.6)
ADI	High Distress (70–100)	949.0 (48.8)
	Moderate Distress (40–69)	486 (25.0)
	Low Distress (1–39)	511 (26.3)
Pack-Years (Mean, SD)		36.51 ± 10.95

Abbreviations: PCP = primary care provider, RUCA = Rural-Urban Commuting Area, SD = standard deviation, ADI = Area deprivation index, LCS = Lung Cancer Screening, LungRADS = Lung Cancer Screening Reporting and Data System

**Table 2:** Unadjusted distribution of low- and high-risk Lung RADS findings by patient characteristics (N = 1880*)

Characteristic	Category	Low Risk Score (N = 1660) n (%)	High Risk Score (N = 220) n (%)	OR (95%CI)	p-value
Age Group	< 65	901 (89.7)	104 (10.3)		
	≥ 65	759 (86.7)	116 (13.3)	1.34 (1.00–1.80)	0.050
Sex	Female	795 (89.5)	93 (10.5)		
	Male	865 (87.2)	127 (12.8)	0.80 (0.60–1.06)	0.117
Insurance	Private	389 (90)	43 (10)		
	Medicaid	388 (89.4)	46 (10.6)	1.07 (0.71–1.62)	0.766
	Medicare	883 (87.1)	131 (12.9)	1.33 (0.93–1.92)	0.124
Race	Black	981 (89.2)	119 (10.8)		
	White	679 (87.1)	101 (12.9)	0.90 (0.67–1.21)	0.157
Indication	Annual	766 (85)	135 (15)		
	Baseline	894 (91.3)	85 (8.7)	1.80 (1.33–2.42)	< 0.001
Provider type	PCP	1420 (88.9)	177 (11.1)		
	Specialist	240 (84.8)	43 (15.2)	0.70 (1.49–0.99)	0.047
Smoking status	Current	1182 (88.2)	158 (11.8)		
	Former	478 (88.5)	62 (11.5)	1.03 (0.75–1.42)	0.850
Area Deprivation Index (ADI)	Low distress	438 (88.7)	56 (11.3)		
	Moderate distress	402 (86.5)	63 (13.5)	1.23 (0.85–1.77)	0.278
	High distress	820 (89)	101 (11)	0.96 (0.69–1.34)	0.817

* Excludes Race categories with small N (Asian and Other) and individuals with missing data.

**Table 3:** Adjusted odds of being in the high-risk category by patient, provider, and place-based characteristics (N = 1880)

	Black (N = 1093)		White (N = 787)		Interaction
Variable	AOR (95% CI)	p-value	AOR (95% CI)	p-value	p-value
**Age (< 65 vs ≥ 65)**	**0.85 (0.58–1.25)**	**0.389**	**0.78 (0.50–1.22)**	**0.276**	**0.213**
Sex (Female vs Male)	0.83 (0.58–1.18)	0.297	0.77 (0.50–1.18)	0.229	0.342
Insurance: Private (ref)	1.00		1.00		
Medicaid	0.89 (0.57–1.38)	0.61	0.84 (0.49–1.44)	0.524	0.408
Medicare	1.16 (0.76–1.77)	0.492	1.28 (0.81–2.03)	0.295	0.312
Indication (Annual vs Baseline)	1.85 (1.32–2.58)	< 0.001	2.10 (1.40–3.15)	< 0.001	0.037
Provider (Specialist vs PCP)	1.32 (0.87–2.01)	0.188	1.58 (1.00–2.50)	0.048	0.058
Smoking Status (Current vs Former)	1.06 (0.72–1.57)	0.776	1.02 (0.63–1.65)	0.927	0.723
ADI: Low (ref)	1.00		1.00		
Moderate	1.20 (0.78–1.85)	0.411	1.27 (0.76–2.13)	0.357	0.452
High	1.09 (0.71–1.66)	0.687	1.15 (0.68–1.95)	0.587	0.384
Pack-Years	1.01 (0.99–1.02)	0.312	1.01 (0.98–1.03)	0.372	0.309

Abbreviations: AOR = adjusted odds ratio, PCP = primary care provider, ADI = Area deprivation index

Low-risk score (reference category- Negative or Benign Lung RADS): No nodules or nodules with benign characteristics. Routine annual screening is recommended.

High-risk score (Probably Benign- requiring a 6-month follow-up CT, or Suspicious Lung RADS - requiring a follow-up within 3 months).

All AORs are adjusted for the following covariates: age group, sex, insurance type, screening indication, provider type, smoking status, Area Deprivation Index (ADI), pack-years with effect modification assessed for race.

Moderate vs High ADI was not significantly associated high-risk scores (p = 0.468).

Statistically significant associations are those with p < 0.05.

Effect modification for race was only significant for screening indication (p-value: 0.037)–the association between screening indication and high risk score was higher in White individuals–and provider type–the association between provider and high risk score was more pronounced and significant in White individuals.

**Table 4:** Adherence to multiple visits for individuals with high-risk scores (N = 220)

Variable	Comparison	OR (95% CI)	AOR (95% CI)	p-value*
Age group	< 65 vs ≥ 65	1.229 (0.632–2.392)	0.852 (0.383–1.896)	0.695
Sex	Female vs Male	1.176 (0.605–2.286)	0.959 (0.472–1.951)	0.909
Insurance	Medicaid vs Medicare	1.412 (0.723–2.759)	1.487 (0.576–3.835)	0.412
	Private vs Medicare	1.412 (0.723–2.759)	1.157 (0.422–3.172)	0.777
Race	Black vs White	1.412 (0.723–2.759)	0.924 (0.432–1.979)	0.839
Smoking	Current vs Former	0.704 (0.347–1.429)	0.687 (0.324–1.458)	0.328
Screening indication	Annual vs Baseline	1.449 (0.718–2.924)	1.338 (0.643–2.786)	0.436
Provider	Specialist vs PCP	0.570 (0.264–1.230)	1.568 (0.687–3.582)	0.285
ADI	Low vs High	–	0.537 (0.214–1.347)	0.185
	Moderate vs High	–	0.393 (0.155–1.001)	0.050
	Moderate vs Low	0.750 (0.342–1.642)	0.72 (0.27–1.89)	0.522
Park-years		1.01 (0.983–1.042)	1.00 (0.976–1.041)	0.441

* p-values for race-based interaction effects

OR: Odds rations, AOR: Adjusted odds ratios (AOR), 95% CI: 95% confidence intervals (CI), PCP: Primary Care Provider, ADI: Area Deprivation Index,

## Data Availability

Data cannot be shared publicly because they contain protected health information from patients within the BJC HealthCare system and Washington University School of Medicine. Data are available from the Washington University Human Research Protection Office (HRPO) (contact: hrpo@wustl.edu) and the Human Data Review Committee (HDRC) (contact: hdrc@wustl.edu) for researchers who meet the criteria for access to confidential data.

## Abbreviations

PCP: primary care provider
RUCA: Rural-Urban Commuting Area
SD: standard deviation
ADI: Areadeprivation index
LCS: Lung Cancer Screening
LungRADS: Lung Cancer Screening Reporting and DataSystem
